# Remote magnetic‐guided ablation for three origins of idiopathic ventricular arrhythmias with right bundle branch block and superior axis

**DOI:** 10.1002/clc.23546

**Published:** 2021-01-20

**Authors:** Xiang Li, Wentao Shang, Ning Zhang, Yun Xie, Yue Wei, Changjian Lin, Tianyou Ling, Kang Chen, Wenqi Pan, Liqun Wu, Yangyang Bao, Qi Jin

**Affiliations:** ^1^ Department of Cardiology, Shanghai Ruijin Hospital Shanghai Jiao Tong University School of Medicine Shanghai China; ^2^ Department of Cardiology Taihe County People's Hospital Hefei China

**Keywords:** catheter ablation, idiopathic ventricular arrhythmia, remote magnetic navigation

## Abstract

**Background:**

Idiopathic ventricular arrhythmias (IVAs) with right bundle branch block (RBBB) and superior axis commonly originate from posterior mitral annulus (PMA), the left ventricular (LV) posterior fascicle (LPF), and the LV posterior papillary muscles (PPM).

**Hypothesis:**

Remote magnetic navigation (RMN)‐guided ablation might be safe and effective for these three origins of IVAs.

**Methods:**

Thirty consecutive IVA patients with RBBB and superior axis (11 MPA‐IVAs, 11 LPF‐IVAs, and 8 PPM‐IVAs) were included in this study. Electrical mapping and ablation with RMN were performed in the LV through a trans‐septal approach. Navigation index, defined as the ratio of total radiofrequency (RF) time and the time from first burn to last burn, was used to determine the efficiency of RMN‐guided ablation.

**Results:**

The overall acute success rate was achieved in 93% (PMA, 100%; LPF, 91%; PPM, 88%; *p* > 0.05). No complication occurred in this study. The procedure time of PPM‐IVAs group was 34 and 14 min longer when compared with MPA‐IVAs and LPF‐IVAs group, respectively, without an increase of X‐ray time. The mean navigation index was 0.45 ± 0.20. The PPM‐IVAs group had an underperforming navigation index value (0.29 ± 0.11) (*p* < 0.01), as longer RF time was required in the PPM‐IVAs group.

**Conclusions:**

RMN‐guided ablation can achieve a high acute success rate for IVAs with RBBB and superior axis. The lower navigation index for PPM‐IVAs indicated that increasing the RF time and improving the catheter contact should be considered when using RMN.

## 1 INTRODUCTION

Idiopathic ventricular arrhythmias (IVAs), including frequently premature ventricular contractions (PVCs) and ventricular tachycardias (VT), originate predominately from the right and left ventricular outflow tracts.^1^ Generally, IVAs have been regarded as non‐lethal arrhythmia,^2^ however, recent studies^3^, ^4^, ^5^ report a subgroup of IVAs with significant mortality, and are a challenge to be successfully eliminated by traditional catheter ablation.^6^ One of the most significant indicators of such arrhythmias is the electrocardiographic (ECG) morphology of right bundle branch block (RBBB) and superior axis. This type of IVA predominately originates from the posterior mitral annulus (PMA), the left ventricular (LV) posterior fascicle (LPF) or the LV posterior papillary muscles (PPM), and rarely from the cardiac apical crux.^7^ Despite some similarities, it is required to distinguish these IVAs from each other, for the differences in prognosis and clinical presentation.^8^ Moreover, in an effort to obtain better ablation outcomes and a shortened procedure time, it is recommended to fully appreciate the precise origin of the tachycardia utilizing algorithms in advance. The remote magnetic navigation (RMN) system has emerged as an alternative to traditional catheter ablation for difficult to treat arrhythmias.^9^, ^10^ Briefly, this robotic system is designed to remotely control the magnetic field generated by the external magnetic poles to guide catheter ablation. Compared with manual operation, it offers precise and flexible catheter maneuver making it easier to reach traditionally challenging target sites. Moreover, reduced radiation exposure and peri‐procedure complications have been observed proving its general safety and broadening its scope of application. Our previous work^11^ has demonstrated the advantages of RMN system in ablation for certain complex arrhythmias. We hypothesize these advantages can be retained on this subgroup. The aim of this study was to investigate the ECG and electrophysiological characteristics and the procedure outcomes of RMN‐guided ablation for IVAs with RBBB and superior axis.

## 2 METHODS

### 2.1 Studied patients

Patients who underwent catheter ablation of symptomatic IVAs with RBBB and super axis in our center, were consecutively included from January 2017 to January 2020. Patients with structural heart diseases including ischemic, valvular, or congenital heart disease were excluded. All patients signed an informed consent before the procedure.

### 2.2 Electrocardiographic analysis

The 12‐lead ECG of VAs was recorded at a paper speed of 25 mm/s in all patients. Analysis of QRS morphology focused on the following characteristics: QS pattern or magnitude of R wave in lead V6; QS pattern or rS pattern in the inferior leads; and monophasic R wave or qR pattern in lead aVR. Waves with a relatively high amplitude (>0.5 mV) were marked with capital letters (Q, R, or S) and vice versa. QRS duration was measured as the interval between the earliest rapid deflections of the ventricular complex in any lead to the latest offset in any lead. The QRS transition zone was determined by the R‐wave amplitude in the precordial leads.

### 2.3 Definition of origin of VA with RBBB and superior axis

The site of origin of the VA was defined by the sites of the earliest ventricular activation or sites of successful ablation established by 3‐dimensional activation map and fluoroscopic images. The PMA region was defined by successful ablation sites located in the LV posterior and posteroseptal region with intracardiac electrogram recordings showing atrial and ventricular potentials during the VA and sinus rhythm. The LPF site was defined by LV posteroseptal region with intracardiac electrogram recordings showing high‐frequency Purkinje potentials preceding QRS onset during the VA and sinus rhythm. The PPM site was defined by anatomic landmarks and successful ablation sites located in the mid‐inferior LV region confirmed by the motion of the magnetic catheter and fluoroscopic images.

### 2.4 Electrophysiological procedure

After withdrawal of antiarrhythmic drugs for more than five half‐lives, the patients underwent electrophysiological study in fasting and conscious state. A deca‐polar catheter and a bi‐polar catheter (St Jude Medical, Inc, St. Paul, MN) were placed within the coronary sinus and at the right ventricle (RV) apex, respectively. A programmed stimulation protocol from multiple RV/LV sites at the 500, and 400 ms drive cycle with up to 3 extra stimuli in decrements down to 200 ms or ventricular refractoriness, was applied to induce VT. Intravenous isoproterenol infusion (1‐10 μg/min) was administered to induce PVCs, if they failed to occur spontaneously.

### 2.5 Mapping and ablation strategy

A transseptal puncture was performed in the LAO radiographic position. Using RMN Niobe™ ES (Stereotaxis Inc, St. Louis, MO) system an open‐irrigated magnetic ablation catheter (NaviStar™ RMT ThermoCool™, Biosense Webster Inc. Irvine, CA) was introduced into the left ventricular cavity using a steerable sheath (MobiCath, Biosense Webster Inc, Irvine, CA.) to perform 3D electro‐anatomic mapping and ablation.

Activation mapping was always performed to identify the earliest activation site of the VAs. We selected potential ablation sites of PVCs where local activation was at least 20 ms pre‐QRS with a QS wave in the unipolar electrogram. Points with QRS morphology during pace‐mapping identical to VAs were also annotated. Entrainment‐mapping techniques were applied trying to characterize the arrhythmic circuit in patients with VTs.

Radiofrequency energy was delivered in the temperature control mode with target catheter temperature of less than 43°C. Power was set at 30‐40 W with a flush rate of 17 mL/min. After catheter ablation, the same stimulation protocol used previously was applied to induce tachycardia. Any induced sustained monomorphic VT was targeted with further mapping and ablation, and the inducible protocol of VT was repeated subsequently until no further VT was inducible. Acute ablation success was defined as the elimination and non‐inducibility of clinical VAs with isoproterenol infusion after at least a 30‐minute waiting period.

### 2.6 Definition of procedural parameters of RMN‐guided ablation

Procedure time was defined as the total time from the Navigant™ (Stereotaxis Inc, St. Louis, MO) ''open procedure'' to the Navigant ''close procedure'', in minutes. Clinical start time was the time at which the catheter registered in the CARTO™ 3D mapping system (Biosense Webster Inc. Irvine, CA) or the time of first applied magnetic field, which ever time was earlier. Clinical time was calculated as the time difference between clinical start time and the latter time of last applied field or last RF ablation application turned off. Mapping time was the time interval from clinical start time to first burn. Total X‐ray time and the control room's X‐ray time were defined as the total sum number of minutes fluoroscopy beam was activated and fluoroscopy beam was activated while magnets were in a navigate position, respectively. RF applications and RF time reflected the total sum number and minutes of ablation burns during the procedure, respectively.^12^ Navigation index, defined as the ratio of total radiofrequency time to the time from first burn to last burn, was utilized to indicate the efficiency of RMN‐guided ablation in this study. The higher the navigation index, the greater percentage of time was spent delivering RF treatment versus locating desired RF treatment locations.

### 2.7 Complications

Complications were divided into two categories: major and minor. Major complications included cardiac tamponade, acute myocardial infarction, stroke, major bleeding. Minor complications were defined as pericarditis and inguinal hematoma.

### 2.8 Follow‐up

Continuous telemetry monitoring was performed for all the patients after the procedure for 24 hours. Patients were then scheduled for outpatient clinic the first 3 months and every 6 months thereafter. The follow‐up 24‐hour Holter recording was performed within 6 months. Recurrence of arrhythmia was defined as either symptomatic recurrence with documented PVCs or asymptomatic frequent PVCs of 5000 per day.^11^

### 2.9 Statistical analysis

The data are expressed as means ± SD for the continuous variables and as numbers and percentages for the categorical variables. Continuous variables were compared using the Mann–Whitney U test, a *p* value <0.05 was considered as statistically significant. All statistical analyses were performed using the SPSS 19.0 (IBM Corp, Armonk, NY).

## 3 RESULTS

### 3.1 Patient characteristics

**FIGURE 1:**
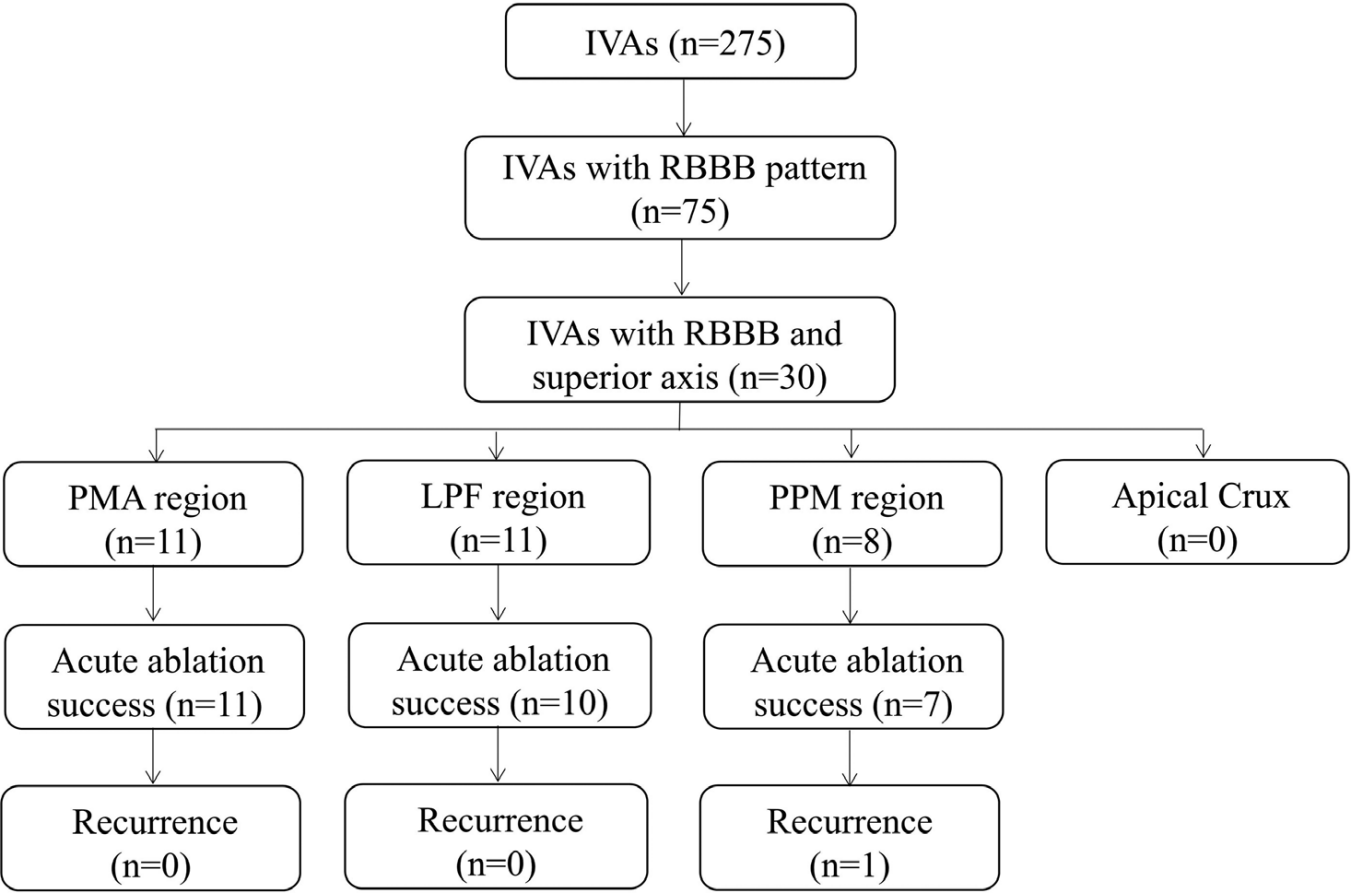
Flow chart of mapping and ablation results. Of the 275 patients who undergo the RMN‐guided ablation for IVAs, 30 patients present with RBBB and superior axis including 11 (37%) with PMA‐IVAs, 11 (37%) with LPF‐IVAs and 8 (26%) with PPM‐IVAs. None has IVA derived from cardiac apical crux. Acute ablation success is achieved in all patients PMA‐IVAs. 10 patients with LPF‐IVAs and 7 with PPM‐IVAs obtain acute ablation success, respectively. Out of 28 patients with acute success, only one patient with PPM‐IVAs recurs without symptom. IVAs, idiopathic ventricular arrhythmias; LPF, left posterior fascicle; PMA, posterior mitral annulus; PPM, posterior papillary muscle; RBBB, right bundle branch block

Of the 275 patients who have undergone the RMN‐guided ablation for IVAs from January 2017 to January 2020 in our center, 30 (11%) had IVAs with RBBB and superior axis, including 11 (37%) with PMA‐IVAs, 11 (37%) with LPF‐IVAs and 8 (26%) with PPM‐IVAs (Figure 1). Their key characteristics were listed in Table 1. The mean patient age was 51.5 years and 83% were male. Patients with LPF‐IVAs were younger (45.1 ± 13.8, *p* < 0.05) and presented more frequently with sustained VT (91% *p* < 0.01) as compared to those with PMA‐IVAs and PPM‐IVAs. No significant difference was detected in gender, hypertension, LVEDd, LVEF, anti‐arrhythmic drugs administration, and VAs burden among the three groups.

**TABLE 1:** Baseline characteristics

	Total (n = 30)	PMA (n = 11)	LPF (n = 11)	PPM (n = 8)	*p*‐value
Age (year)	51.5 ± 16.0	57.9 ± 12.6	45.1 ± 13.8	51.4 ± 20.8	<0.05
Gender (male, %)	25 (83%)	8 (73%)	11 (100%)	6 (75%)	>0.05
Hypertension (%)	13 (43%)	7 (64%)	3 (27%)	3 (38%)	>0.05
LVEDd (mm)	49.8 ± 6.0	51.4 ± 8.7	49.5 ± 3.1	48.1 ± 4.9	>0.05
LVEF (%)	64.8 ± 5.5	64 ± 8.1	63.5 ± 2.3	67.5 ± 4.1	>0.05
≥1 AADs (%)	26 (87%)	8 (73%)	10 (91%)	8 (100%)	>0.05
Clinical VAs manifestation					
Sustained VT (%)	15 (%)	1 (9%)	10 (91%)	4 (50%)	<0.01
PVC (%)	15 (%)	10 (91%)	1 (9%)	4 (50%)	<0.01
VA burden (thousands/24 h Holter)	23.8 ± 9.6	22.9 ± 10.5		27.3 ± 8.5	>0.05
ECG manifestation					
Duration of QRS complex (ms)	143 ± 12	148 ± 9.6	132 ± 7.0	153 ± 9.9	<0.01
R or RS pattern in I (%)	18 (60%)	11 (100%)	6 (55%)	4(50%)	<0.01
Precordial transition at V3	11 (33%)	0 (0%)	6 (55%)	5 (63%)	<0.001
rS pattern in V6 (%)	19 (63%)	0 (0%)	11 (100%)	8 (100%)	<0.001
Electrophysiological manifestation (target potential)					
A & V potentials (%)	11 (37%)	11 (100%)	0 (0%)	0 (0%)	<0.001
Purkinje potentials (%)	16 (53%)	0 (0%)	10 (91%)	6 (75%)	<0.001

Abbreviations: AAD, anti‐arrhythmic drug; A & V, atrial and ventricular; ECG, electrocardiogram; LVEDd, left ventricular end diastolic diameter; LVEF, left ventricular ejection fractions; PVC, premature ventricular contraction; VA, ventricular arrhythmia; VT, ventricular tachycardia.

### 3.2 ECG and electrophysiological findings

**FIGURE 2:**
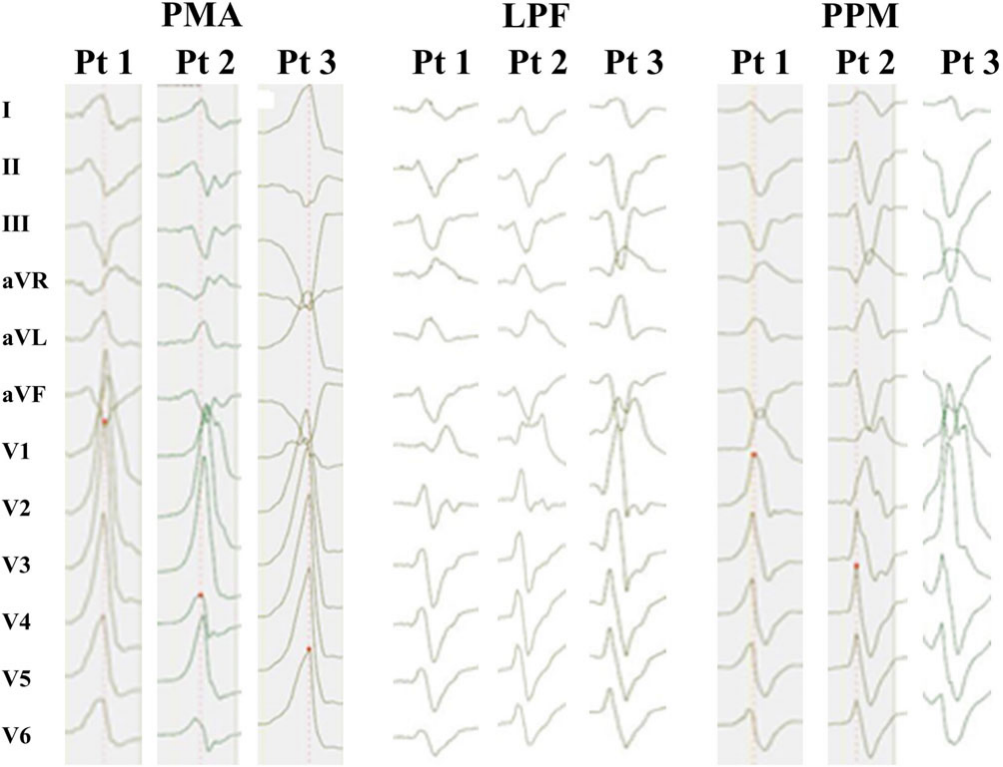
Representative 12‐lead ECGs of IVAs with RBBB and superior axis. IVAs, idiopathic ventricular arrhythmias; LPF, left posterior fascicle; PMA, posterior mitral annulus; PPM, posterior papillary muscle; RBBB, right bundle branch block

For each group, ECG manifestation demonstrated a morphology of RBBB and superior axis during onset of IVAs (shown in Figure 2). Duration of QRS complex varied. Patients with PPM‐IVAs presented with the widest QRS complex, followed by PMA‐IVAs and LPF‐IVAs cohort (153 ± 9.9 ms vs. 148 ± 9.6 ms and 132 ± 7.7 ms, *p* < 0.01). There were considerable differences of R/RS pattern presence in lead I. All patients with PMA‐IVAs showed a R or RS pattern, whereas only almost a half of patients with LPF‐IVAs and PPM‐IVAs presented with this pattern (*p* < 0.01). Similarly, precordial transition zone occurred in lead V3 for roughly half of patients with LPF‐IVAs and PPM‐IVAs, however no precordial transition zone was observed for PMA‐IVAs cohort (*p* < 0.01). The result of rS pattern presence in lead V6 fell into two groups: all patients with LPF‐IVAs and PPM‐IVAs presented with it, while patients with PMA‐IVAs did not (*p* < 0.01).

Electrophysiological manifestation varied considerably for each group. Patients with PMA‐IVAs all presented with far‐field atrial potentials and ventricular potentials whereas none presented with these potentials in the LPF‐IVAs and PPM‐IVAs cohorts. Additionally, no patient with PMA‐IVAs presented with purkinje‐like potentials (*p* < 0.01). ECG and electrophysiological manifestation were listed in Table 1.

### 3.3 Acute success and procedural outcomes

**FIGURE 3:**
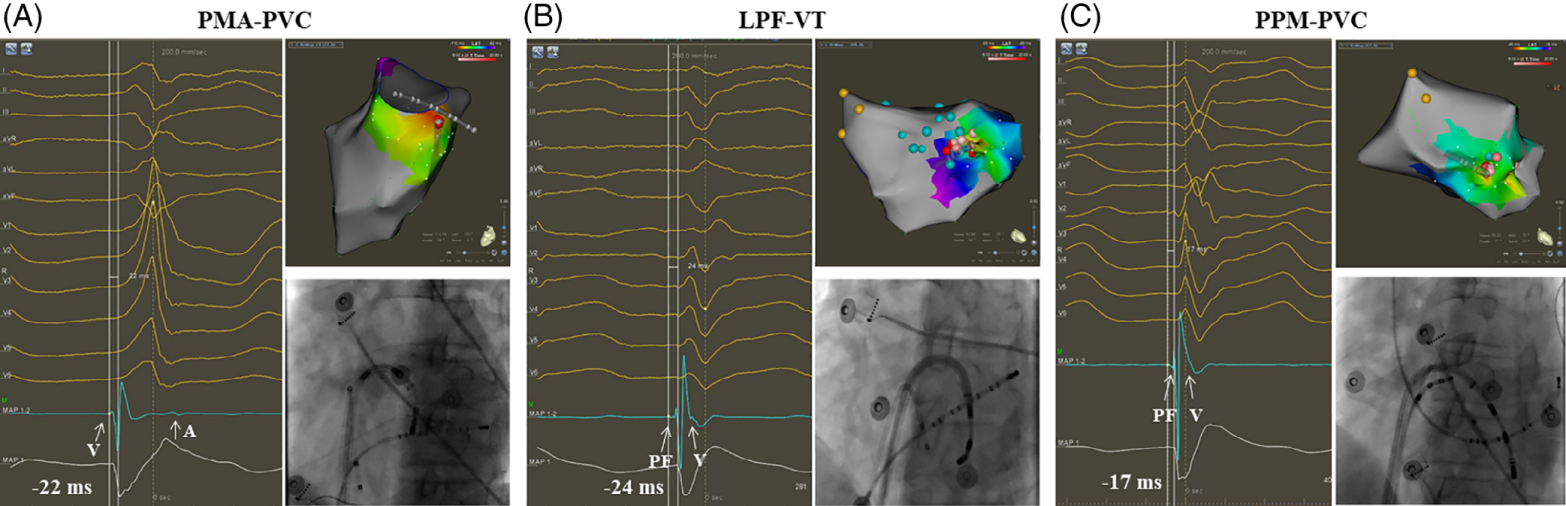
Examples of successful ablation of IVAs with CRBBB and superior axis. The catheter is pointing at the site with the earliest activation time prior to QRS onset through a trans‐septal approach in each panel. The fluoroscopic images on the bottom right corner of three panels show the catheter positions of the target ablation points. Panel A shows an example of PMA‐PVC ablation: A far‐field atrial potential (''A'' arrow) can be recorded. Panel B shows an example of LPF‐VT ablation. Blue points indicate the fascicular potentials. A purkinje potential prior to ''V'' potential can be recorded. Panel C shows an example of PPM‐PVC ablation. Yellow points indicate the ''His'' potentials. A purkinje potential prior to ''V'' potential can be recorded. LPF, left posterior fascicle; PMA, posterior mitral annulus; PPM, posterior papillary muscle; PVC, premature ventricular contraction; RBBB, right bundle branch block; VT, ventricular tachycardias

Overall, the mean acute success rate was achieved in 93%. Examples of successful ablation of IVAs with CRBBB and superior axis is shown in Figure 3. Although, no significant difference was observed in ablation acute success rate (PMA, 100%; LPF, 91%; PPM, 88%; *p* > 0.05), there was a downward trend for patients with PPM‐IVAs. Procedure outcomes of 30 enrolled patients are detailed in Table 2. The mean procedure time, clinical time and mapping time were 89.2 ± 38.6 mins, 57.5 ± 36.5 mins, and 25.2 ± 11.4 mins, respectively. Total X‐ray time and control room's X‐ray time were 4.2 ± 2.4 mins and 0.76 ± 0.72 mins, respectively. RF applications and RF time were 11.8 ± 11 and 8.8 ± 6.4 mins. Procedure time and RF time of patients with PPM‐IVAs were longer, compared to those with PMA‐IVAs and LPF‐IVAs (Table 2). While no significant difference was observed in total X‐ray time and control room's X‐ray time between groups. Regardless of the encouraging average score of navigation index in all groups (0.45 ± 0.20), patients with PPM‐IVAs underperformed the index (0.29 ± 0.11) (*p* < 0.01) (Table 2).

**TABLE 2:** Procedural outcomes and acute success

	Total (n = 30)	PMA (n = 11)	LPF (n = 11)	PPM (n = 8)	*p*‐value
Procedure time, mins	89.2 ± 38.6	72.7 ± 34.9	92.8 ± 42.9	107 ± 31.7	<0.05
Clinical time, mins	57.5 ± 36.5	40.4 ± 27.8	63.9 ± 43.9	72.3 ± 30.1	<0.05
Total X‐ray time, mins	4.2 ± 2.4	4.3 ± 2.8	4.2 ± 2.3	3.9 ± 2.1	>0.05
Control room X‐ray time, mins	0.76 ± 0.72	0.66 ± 0.45	0.62 ± 0.54	1.1 ± 1.1	>0.05
RF applications, n	11.8 ± 11	8.1 ± 4.7	11.6 ± 13.3	17.0 ± 12.8	<0.05
RF time, mins	8.8 ± 6.4	6.7 ± 4.3	9.6 ± 8.9	10.5 ± 4.3	<0.05
Mapping time, mins	25.2 ± 11.4	17.8 ± 5.2	30.3 ± 10.3	28.3 ± 14.3	<0.01
Navigation index	0.45 ± 0.20	0.50 ± 0.21	0.51 ± 0.20	0.29 ± 0.11	<0.05
Acute success rate, n (%)	28 (93%)	11 (100%)	10 (91%)	7 (88%)	>0.05

Abbreviation: RF, radiofrequency.

### 3.4 Complications and follow‐up

The average follow‐up duration was 22.1 ± 5.9 months after the procedure. No procedure‐related complications occurred during ablation or follow‐up. Out of 28 patients with acute success, only one patient with PPM‐IVAs recurred without symptom.

## 4 DISCUSSION

### 4.1 Major findings

This is the first study to evaluate the efficacy and safety of RMN‐guided ablation for IVAs with RBBB and superior axis in a series of patients. The main findings are as follows. ECG and electrophysiological characteristics can identify the origin sites of IVAs with RBBB and superior axis. RMN‐guided ablation can achieve a high acute success rate (93%) for IVAs with RBBB and superior axis without procedure‐related complication. The lower navigation index for PPM‐IVAs indicated that increasing the ablation time and area, and improving the catheter contact should be considered when using RMN.

### 4.2 ECG and electrophysiological characteristics of VAs arising from PMA, LPF, and PPM

In order to obtain optimal procedure efficacy and shortened procedure and X‐ray time, it is crucial to precisely understand the origin site to specify treatment strategy prior to the procedure.^13^, ^14^ QRS complex duration may be a potential way to distinguish LPF‐IVAs from PPM‐IVAs. The relatively posterior exit location^15^ and conduction delay through the PPM into the normal myocardium^8^ probably accounts for the duration difference. Yamada^16^ et al reported a QRS duration >160 ms as the only predictor for distinguishing PPM‐IVAs from LPF‐IVAs. Though a significant QRS complex duration difference was observed in this study (153 ± 9.9 vs. 132 ± 7.0, *p* < 0.01), few patients with PPM‐IVAs in this study presented with a>160 ms QRS duration. Variability of anatomical location and limitation of sample size may explain the difference in results. Given that this indicator is still controversial, we explored other ECG and electrophysiological characteristics. Occurrence of purkinje potentials could identify LPF‐IVAs from PPM‐IVAs with a 91% sensitivity and 68% specificity. Different from LPF‐IVAs and PPM‐IVAs, all the targets of PMA‐IVAs had the electrophysiological manifestation of far‐field atrial and ventricular potentials.

### 4.3 Advantages and best practices of RMN‐guided ablation

Catheter ablation for IVAs with RBBB and superior axis in this study were all guided by RMN. It is challenging for manual catheter ablation to eliminate this subgroup of IVAs, due to difficulty maneuvering the catheter to these locations. However, under the guidance of RMN, precise and flexible catheter navigation can be relatively easy to obtain,^17^, ^18^ when compared to manual catheter ablation for complex arrhythmias. One study^19^ reported the result of manual catheter ablation for 8 patients with PPM‐IVAs to be an acute success rate of 87.5%, which was similar to our results (88%). However, the manual catheter study's recurrence rate was 85.7%, which is much higher than the current study (14%). This is likely related to the inherent limitations of manual pull‐wire catheter manipulation.^20^ Deeper tissue sites can be a source of certain PPM‐IVAs which demands for sufficient stable contact of catheter to tissue. PPM‐IVAs can also arise from the tip of PM^21^ which makes it difficult to maintain flexible and precise stability in this area during manual catheter ablation. With a magnetic catheter, the interaction between the applied external magnetic field and catheter's internal magnets allows for precise distal tip movements and catheter stability, making it possible to achieve navigation requirements for this type procedure. RMN‐guided ablation allows for stable, focal contact between tissue and catheter, which could explain the relatively low recurrence rate in the current study. Although no patients with cardiac apical crux‐IVAs have been encountered, it is likely that the attributes of RMN would be as effective and safe for ablating such subgroup of IVAs in view of the current high success rate in this study.

### 4.4 Implications of procedural outcomes of RMN‐guided ablation

Overall procedure time (89.2 ± 38.9) and total X‐ray time (4.2 ± 2.4) were remarkably low in this study. PPM‐IVAs procedure parameters that included procedure time, clinical time, mapping time and RF time were all longer when compared with LPF‐IVAs and PMA‐IVAs procedure times. This might be related to the anatomy heterogeneity and complexity of papillary muscles.^10^ Interestingly, the longer procedure time did not extend X‐ray time. In fact, no significant difference was observed in total X‐ray time and control room's X‐ray time in this study. In this study, a novel parameter (navigation index) was utilized to assess the accuracy and efficiency of the ablation protocol. The navigation index of PPM‐IVAs ablation was much lower than either LPF‐IVAs or PMA‐IVAs in this study, indicating that increasing the RF time and area should be considered when using RMN for PPM‐IVAs. For all that, however, we have to acknowledge the inherent advantages of RMN as verified by reduced X‐ray time and low complication and recurrence rate. Cryoablation has been proposed to meet the stability of catheter in ablation for PPM‐IVAs, while this comes with its own limitation in flexibility and ablation depth.^22^ Circumferential PM ablation is another direction^23^ and this high demand for catheter control is also a chance for RMN‐guided ablation to shows its fascinating advantages.

## 5 LIMITATIONS

Our impressive initial report on RMN guided‐ablation for IVAs with RBBB and superior axis needs to be further confirmed by larger randomized controlled trials. A more accurate mapping and ablation procedure could be facilitated by combing intracardiac ultrasound with RMN. IVAs arising from apical crux, as rare cases, were not enrolled in this study. Further study may demonstrate if ablation strategies employed here can be successfully applied to this type of IVA as well.

## 6 CONCLUSIONS

RMN‐guided ablation for IVAs with RBBB and superior axis achieved a high acute success rate in most selected patients, proving its efficacy and safety in these cases. The lower navigation index for PPM‐IVAs indicated that increasing the RF time and improving the catheter contact should be considered when using RMN.

## CONFLICT OF INTEREST

The authors declare no potential conflict of interest.

## Data Availability

The data of this study are available from the corresponding author upon request.
